# Dental Education—Office Pupilage

**Published:** 1890-02

**Authors:** J. Taft


### THE DENTAL REGISTER.

VOL. XLIV.] FEBRUARY, 1890. [No. 2.

### COMMUNICATIONS.

### Dental Education—Office Pupilage.

J. TAFT.

Before the establishment of dental colleges there were two methods of entering the practice of dentistry. The one was adopted by those who had, or imagined they had, a natnral indowment or ability, which by a little practical experience and observation would enable them to accomplish all that was required or expected of the dentist by the public.

These were the so-called (and often self-styled) self-made dentists. They often set a high estimate upon their manipulative talent and skill, and finding the door open and the way clear after obtaining a few instruments, appliances, and materials, they would announce themselves by sign, advertisement, and boasting as prepared and ready to do all that dental art and skill could accomplish.

They placed great stress upon the fact that they made their own instruments, and they endeavored to impress those with whom they came in contact that there was much of mystery and things difficult of comprehension in their practice. This class of dentists, and this method of entering the practice, fortunately have now well nigh passed away.

The other method of preparation for dental practice was by a system of office instruction which consisted in the personal teaching of the student by the practitioner. This was usually by a definite and well-arranged contract in which both parties engaged to perform and fulfill certain duties. Those on the part of the practitioner were to impart to the pupil a knowledge of the science and art of his profession so far as he might be able.

The faithful instructor also regarded himself as bound to set before his pupil, both by precept and example, the *ethical qualities* that should attach to every one who assumes so responsible a calling as the practice of dentistry.

The student on his part engaged to give strict attention to the work in hand, to follow closely the suggestions and instructions of his preceptor. He was also to render to his teacher a stipulated consideration; this was oftentimes in money, either wholly or in part. Service that the pupil might be able to render was sometimes accepted as part of the consideration. Such pupilage was usually continued for from one to three years. In many such cases the pupils were either graduates or students of medicine, and of such have been some of the most renowned men of our profession; for example, C. A. Harris, E. Parmley, Solymon Brown, E. Carr, H. H. Hayden, E. Maynard, J. F. Flagg, Daniel Harwood, E. G. Tucker, Joshua Tucker, Ewd. Hendson, Ewd. Taylor, Jas. Taylor.

After the establishment of dental colleges another and broader way was opened for entering the practice of dentistry. This, however, did not at once, nor has it yet, wholly superceded the two methods above referred to, though very rarely now will any one venture to assume the responsibility of the dentist without some systematic course of instruction under the direction of a qualified teacher, and even the instances in which this is accepted as a means of full preparation are now rare. Indeed, so broad is the curriculum for the proper equipment of the dentist that no single teacher can afford the time, nor can be adequately qualified to impart the requisite instruction in all branches now regarded as important and necessary.

This brings us to the chief inquiry of this paper, viz.:

With the opportunities and facilities that now exist for scholastic dental education and training, *is an office pupilage, or private instruction for the best interest of the student*, as preparatory to his college course?

In giving an answer to this question there are so many circumstances and conditions to be taken into account that an unquestioned solution is hardly within our reach. But after all it is a question of moment, one the importance of which we can not ignore. Opinions both affirmative and negative have often been expressed.

Frequently the basis or ground for these opinions falls short of a full comprehension of all that pertains to the subject. Private pupilage will be beneficial to the student under the following circumstances, viz.: A good teacher, which embraces the following things:

1st. One who fully *understands* that which he is to teach.

2nd. The ability to *readily and well communicate* what he *knows*.

3rd. The ability of inciting in the mind of his pupil an appreciation and love for his profession.

4th. One who by precept and example will lead his pupil in a path of high moral rectitude, and inspire him with the instincts of the true gentleman.

Conditions should attach to him who is to be taught, as well as to the teacher:

1st. He should be adapted by natural endowment to the work upon which he is to enter, and this embraces not only manipulative skill, but a love for, and a persevering industry in his work.

He should possess, at least, a good English education, and to this it is very desirable that a good degree of scientific and classical knowledge be added.

3rd. He should be energetic and persevering in whatever he undertakes.

4th. He should possess high moral and ethical principles.

It is conceded that there are many instances in which these requirements will not be all realized; but it is well to set up a good standard and work as nearly to it as possible.

Whenever private or office dental pupilage is attended by favorable conditions and circumstances good results (in some respects) are sure to follow, and in the first place the student is placed in a professional path from which he is not likely to deviate.

Second, the influence of the correct and efficient private teacher is greater and more permanent than that of a public teacher, and mainly because of more intimate professional contact. His instructions, if faithful, will be more enduring.

He who has had a good private instructor will often reflect the characteristics of the latter through life, while rarely, if ever, will this be true of the public instructor, at most, only to a limited degree.

The private teacher can, and usually does, reproduce his own professional traits and characteristics in his pupil, and this in a marked degree under favorable conditions.

The question whether under the present advanced and efficient scholastic methods of instruction, private pupilage, even of a preliminary kind, is desirable, or for the best interests of the student, is one upon which different opinions are entertained. But with a good and faithful preceptor a student can, in from one to two years be so prepared, that the systematic college course will be of much greater value to him than if he had entered the school without such preparation.

The question whether by careful selection and thorough training by a private instructor, within a reasonable sphere or limit, prior, and in addition to the college course, the educational work preparatory to the practice of dentistry, would not bring forth better results, is one worthy of special consideration.

The system of apprenticeship, as practiced in England, we may do well to consider before settling to a conclusion on this subject.

Under this system a student is apprenticed for from three to four years, and during this time the student is confined in the main, to the study and working out of mechanical principles and processes. this in certain respects fitting him, in a good degree, for his future work.

Will not a well-directed and properly-arranged pupilage under a private preceptor, in addition to the college course, give better results than the indiscriminate admission to our colleges of those wholly innocent of any knowledge of dental art or science ?

